# No man’s land support the endemic Red Sea ghost crab (*Ocypode saratan*) in the Gulf of Eilat

**DOI:** 10.1038/s41598-024-63326-y

**Published:** 2024-05-31

**Authors:** Reuven Yosef, Royi Elharar, Jakub Z. Kosicki

**Affiliations:** 1 Ben Gurion University of the Negev - Eilat Campus, P.O. Box 272, 88000 Eilat, Israel; 2 Rabin High School, Yotam Street 51, 88104 Eilat, Israel; 3 Department of Avian Biology and Ecology, Faculty of Biology, Adam Mickiewicz University, Poznań, ul. Umultowska 89, 61-614 Poznań, Poland

**Keywords:** Tourism, Eilat, Red sea, Endemic, *Ocypode saratan*, Conservation biology, Freshwater ecology

## Abstract

Tourism pressure on the Red Sea ecosystem have posed significant threats to numerous endemic species, including the Ghost Crab *Ocypode saratan*, which is exclusively found along a small stretch of beach in the Eilat/Aqaba Red Sea Gulf. Due to the limited understanding of their ecology, we investigated how tourism impacts the behavior of this species. Employing a natural setup, we compared burrow dimensions, pyramid structures, and density across three distinct beach sections subjected to varying levels of human interference. Access to a secluded beach, referred to as “No Man’s Land,” provided a crucial control for our study. This facilitated a comparative analysis of ghost crab activity among beaches experiencing differing levels of human disturbances: (1) a tourist beach characterized by continual high disturbance, (2) a naval beach subject to moderate and sporadic disturbances, and (3) the isolated “no man’s land” beach devoid of human presence. Our observations revealed notable differences in ghost crab density among the three beaches. Furthermore, we observed that on the secluded beach, larger individuals tended to establish burrows farther from the waterline and construct taller sand pyramids. Given the significance of sexual selection processes, their conservation becomes imperative for the survival and potential expansion of the ghost crab population across the Gulf of Eilat/Aqaba. We propose a straight-forward and cost-effective strategy: the designation of short, secluded beach enclaves along this gulf. We believe that this approach will mitigate adverse impacts of tourisms while simultaneously benefiting various sandy beach species.

## Introduction

In the current Anthropocene era, characterized by widespread human influence across nearly all ecosystems on Earth, a primary focus of conservation biology lies in assessing the adaptability of wild species to degraded environments^1,2^. While some posit that animals can adjust their behaviors to accommodate human presence and activities^2,3^, the ramifications of such adaptations extend beyond mere behavioral changes^4^. They encompass alterations in temperament, shifts in social structures, and impacts on the recruitment of young animals into breeding populations^5–7^.

Despite their crucial role, scavengers, often associated with human activities, are frequently undervalued^1,8^. These organisms primarily provide ecosystem services, notably contributing to public health by controlling diseases through the removal of carcasses and organic material from human environments^9^. While vertebrate carnivores are commonly recognized for providing such services^1,8,9^, invertebrates such as ghost crabs (*Ocypode* spp.) also play a crucial role in maintaining environmental quality. Particularly in seaside resorts where organic waste accumulates on sandy beaches, ghost crabs are essential for its removal and contribute to overall ecosystem health^10,11^.

The endemic Red Sea ghost crab (RSGC; *O. saratan*) remains a poorly understood species, with knowledge gaps regarding its ecology and distribution^12^. Early studies were conducted in Egypt and Saudi Arabia during the mid-twentieth century^13–16^, but research on the species was neglected until recent projects in Eilat, Israel^17–19^. Despite extensive tourist development along its beaches, a small portion of the original habitat in this resort town has been partially preserved, providing a refuge for a remnant population of RSGC.

Surveys conducted along the Aqaba beach in Jordan failed to locate any RSGC on the Jordanian side of the Gulf of Aqaba/Eilat, suggesting that the last inhabited fragment of habitat for this species within the Gulf lies along a 447.7-m stretch of beach, specifically in Eilat. Previous studies here have primarily focused on the tourist section ^17,18^ or comparisons with the adjacent naval section ^19^. Interestingly, the tourist section attracts a higher number of ghost crabs, while section of beaches experiencing intermittent human disturbances, such as military activities, exhibit higher pyramid structures. Since these structures play a significant role in sexual selection, it is speculated that beaches with limited human interference serve as primary breeding grounds^17^. However, it remains unclear whether solely restricting human interference on specific beach sections is adequate for sustaining populations of the Ocypode genus. To address this question, additional experimental groups should be included, encompassing sections of beach entirely devoid of human activity. By comparing the abundance and behaviors of the crabs between the naval beach (with limited human access) and a beach devoid of any human interference, insights can be gained into the species’ response to varying levels of human pressures. Such findings could bear significant implications for formulating conservation strategies aimed at protecting endemic coastal species.

For the first time, we obtained permission to access the closed section of beach located east of the naval facility, representing a No Man’s Land area along the international border with Jordan. This presented us with a unique opportunity to conduct a study comparing the behavioral parameters of the RSGC across beaches with differing levels of human disturbance. These included (1) the tourist beach, characterized by the highest and continuous disturbance, (2) the naval beach, subject to moderate but sporadic disturbance, and (3) the no man’s land beach, inaccessible to humans. This natural experiment presented a unique opportunity to evaluate the influence of tourism on RSGC activity. Comparative studies have demonstrated that human activities can exert detrimental effects on other *Ocypode* species. For instance, sand replacement and mining caused population declines of *O. quadrata* in Brazil and Ghana^10,20^. Ecotourism has halved the number of ghost crabs on urban beaches in Australia^21^ and affected *O. ceratophthalmus* population in Singapore^22^. Moreover, recreational vehicles on subtropical beaches in southeast Queensland, Australia, have led to declines in *O. cordimanus* populations^23^.

Through conducting a quantitative analysis within the natural field experiment, our aim was to explore the effect of tourist pressure on RSGC activity within the last remaining habitat of Gulf of Eilat/Aqaba. We hypothesized that a closed section of beach without human interference would function as an enclave, attracting a higher number of individuals, with larger ones having greater competitive abilities. Additionally, we expected that the absence of tourist pressure would result in taller sand pyramid structures, serving as a secondary sexual signal^17^, and that burrows in undisturbed habitats would be situated further up the beach from the waterline.

## Material and methods

**Figure 1:**
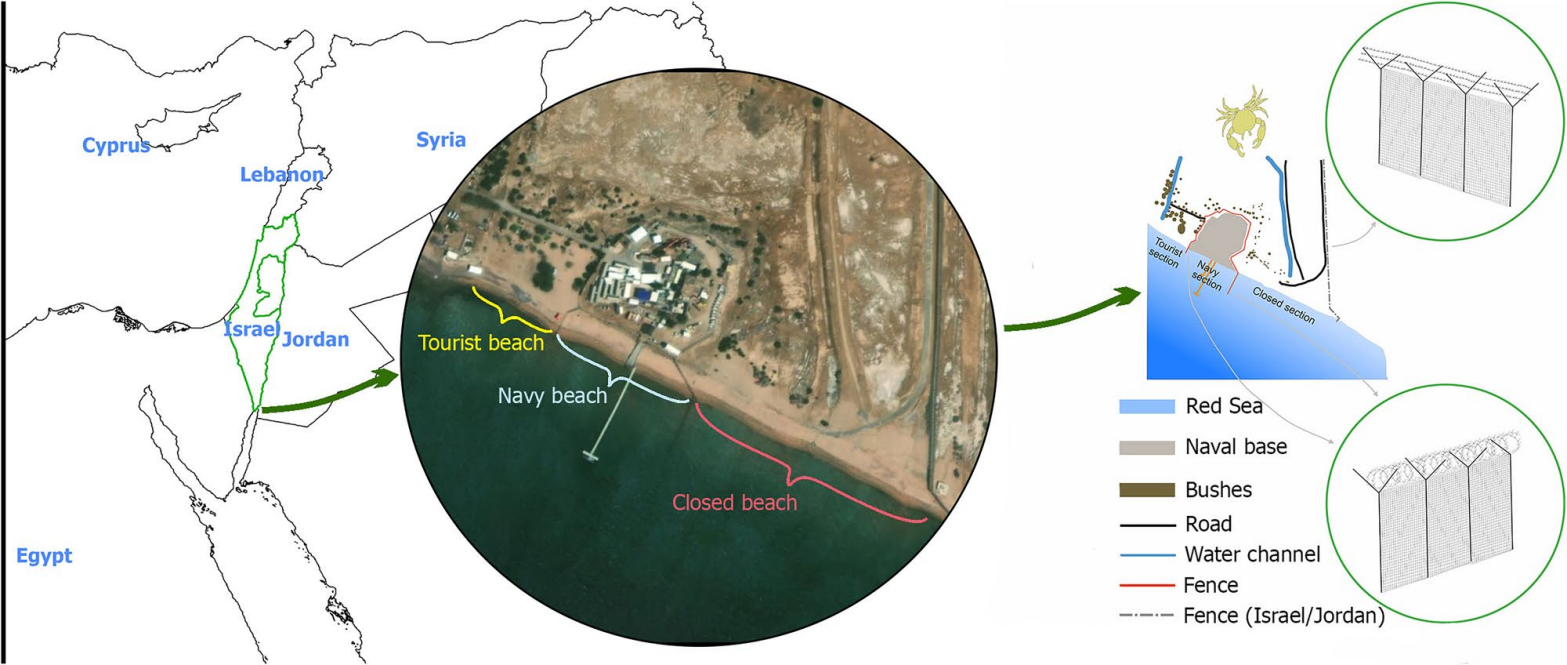
Image of the study area in the eastern part of the North Beach of Eilat, Israel. Note that in the satellite photo from Google Earth 10.49.0.0. (https://earth.google.com/web/) marked beaches are sandy, while to the west the gray gravel stands out. The attached overview map was made by the author (JZK) based on field inspections and using QGIS 3.16 (https://qgis.org/pl/site/forusers/download.html) and GIMP 2.10 (https://www.gimp.org/).

The study was conducted on the northeastern beach of the Israeli side of the international border with Jordan. The beach is comprised of three sections forming a continuum from each other (Fig. 1), although they are divided into three unequal sections (each separated by a fence) due to their proximity to the international border. The first section, the closed beach (29.542898° N, 34.977426° E), extends from east to west and is strictly off-limits to the public without special permission and supervision, as it forms part of the No Man’s Land along the international border with Jordan. This beach measured 240.6 m in length covering a study area of 4606.0 m^2^. Adjacent to the closed beach section to the west is a naval facility (29.543853° N, 34.975158° E), also inaccessible to the public. This section measured 125.9 m in length, covering an area of 2327 m^2^. Activity on this beach is sporadic and primarily occurs during naval exercises or emergencies, categorized as moderate human use .

Further west of the naval facility is the tourist beach (29.544174° N, 34.974473° E), spanning 81.2 m in length and covering an area of 2855.6 m^2^. This beach experiences intensive usage day and night by campers, recreationists, and tourists. Initially fenced off until 2009 as a logistics area for the fish farms. The Eilat municipality declared this section of beach as public after the dismantling of the controversial aquaculture. Consequently, all three study sections represent the last remaining pristine beaches in Eilat, retaining their natural sea sand. Unlike the rest of the local beaches, which were covered with quarry gravel or concrete/wooden boardwalks to enhance tourist accessibility, these study sections retained their natural state.

To calculate the RSGC density at each beach, we initially determined the length of the waterline at high tide for each beach section by measuring the total shoreline length during high tide. We quantified the number of active crabs as the count of individuals moving between their burrow and the water during the study period. To prevent counting the same individual multiple times during each beach inspection, we conducted counts once over a 10-min interval. We then calculated the crab density (CRABS) for each inspection by dividing the number of active individuals by the area of the respective beach section.

The study was conducted over a period spanning from March 7 to June 12, 2022. Access to the closed section of the beach was granted for seven full-day visits. Simultaneously, evaluations were also conducted at the other two sections, ensuring that all data were collected within several hours. Due to military restrictions, visits were limited and required entry into the naval base and No Man’s Land one at a time. The visit dates were as follows: March 7 and 31, April 3 and 28, May 6 and 12, and June 12, 2022. In total, 158 working hours were spent collecting data across the three sections, averaging 22.6 h/day with permitted access.

We focused solely on occupied burrows, identified by signs of activity such as fresh tracks in the sand or visible movements of crabs at the burrow entrance^17–19^. For each occupied burrow, we measured both its height and length, corresponding to the carapace size of the resident crabs (ENTRANCE), drawing upon established methodologies^24–26^. Additionally, we recorded the distance between each burrow and the waterline (DISTANCE) at high tide (https://www.tide-forecast.com/locations/Eilat/tides/latest) to understand habitat preferences. In cases where a courtship pyramid was present near the burrow, we also measured its height (PYRAMID).

The daily hours were categorized into three segments: morning (8:00–10:30), afternoon (12:00–15:00), and evening (18:00–21:00). We chose these time intervals based on prior research, which highlighted the study species diverse diurnal activity patterns. Subsequently, we integrated these time segments into our models to accomodate potential fluctuations in activity levels throughout the day.

## Statistical analysis

We conducted an analysis to compare the size of burrows (ENTRANCE), the height of pyramids (PYRAMID), the distance between burrow and the waterline (DISTANCE), as well as the density of the crabs (CRABS) among the three sections of the beach, each experiencing varying levels of human activity. We assumed that the size of the burrow entrance could serve as a proxy for the relative size of the individual inhabiting it, i.e., the carapace width ^14,24,25,27,28^. The size of the burrow entrance was determined by calculating the area of the ellipse using the following equation:

$$V = \frac{\pi \times A \times B}{4}$$

where π ~ 3.14, A—width of the ellipse, B—height of it.

Our four variables, namely the height of the pyramid (PYRAMID), distance between the burrow and the waterline (DISTANCE), the number of active individuals (CRABS), and the size of the entrance (ENTRANCE), showed no significant correlations with each other (*p*-value for Spearman’s correlation > 0.1 in all cases, Fig S1).

To test the differences between the dependent variables across the three different sections of the beach, we initially employed a Kruskal–Wallis test. However, we observed significant differences between study days for PYRAMID, ENTRANCE, and DISTANCE (with all *p*-values < 0.001, no difference for CRABS, details see Appendix), as well as differences across time of day (with all *p*-values < 0.001, details see Appendix).

To address the factors affecting our dependent variables (H.PYRAMID, DISTANCE, CRABS, ENTRANCE), we developed Generalized Linear Mixed Models (GLMMs). These models incorporated beach types (closed, naval, and tourist) as the grouping variable, while study days and time of day were treated as random factor intercepts. This approach allowed us to analyze the differences in four parameters describing crab activity while simultaneously controlling for factors resulting from methodological constraints. For PYRAMID and DISTANCE we used a normal distribution (see Fig. S2A-B) while for ENTRANCE and CRABS we used a Gamma distribution (see Fig. S2C-D). In GLMM, categorical variables are encoded using a “one-hot” coding scheme, where one category is selected as the reference (base) category and does not appear directly in the model results as a separate coefficient. In our study, the “closed” beach was selected as the reference category. Therefore, the coefficients for the “naval” and “tourist” beaches indicte the difference in the expected value of the dependent variables (PYRAMID, DISTANCE, CRABS, ENTRANCE) relative to the closed beach. GLMMs with an independent variable as a factor with three levels make it impossible to compare variables between the tourist and naval beaches. To address this, we conducted a contrast analysis for each model. Additionally, we calculated both marginal and conditional pseudo-R^2^ values. The marginal pseudo-R^2^ represents the percentage of variance explained by the fixed predictors, while the conditional pseudo-R^2^ represents the percentage of variance explained by the entire model, including both fixed and random effects.

We conducted all calculations in R using the libraries lme4^29^, lmerTest^30^, emmeans^31^, and performance^32^.

## Ethics

Study based on observations. No animal handling or relevant permits required. Access to the naval base and no-man’s-land was courtesy of the officers of the Israeli Navy.

## Results

We recorded 805 burrows: 463 (57.5%) on the closed section of beach, 262 (32.5%) on the naval section and 80 (9.9%) on the tourist section. The mean (SD) density of RSGC on the three sections was 0.02 ± 0.04 individuals/m^2^ for the tourist section, 0.11 ± 0.02/m^2^ for the naval section, and 0.10 ± 0.02/m^2^ for the closed beach. Statistical analyses revealed no significant difference in density among the three beach sections (chi-square = 0.06, *p* = 0.968).

Based on the results of the Kruskal–Wallis test (Table 1), we observed that courtship pyramids (PYRAMID) and entrance area (ENTRANCE) were biggest on the closed section, while the smallest entrance with the lowest pyramids were observed on the tourist section of beach. Similarly, we found that the majority of the crabs (DENSITY) with burrows furthest from the waterline (DISTANCE) were on the closed section of the beach (Table 1).

**Table 1:** The mean number of Red Sea ghost crabs (*Ocypode saratan*) of various sizes and their activity on tourist, naval and closed beaches.

	Tourist beach				Navy beach				Closed beach				Kruskal–Wallis	
	Mean ± SE	Median ± mad	N	range	Mean ± SE	Median ± mad	N	range	Mean ± SE	Median ± mad	N	range	H*	*p*
Pyramid	11.64 ± 1.99	11.50 ± 2.22	80	8–15	14.92 ± 2.11	15.00 ± 2.22	262	10–19	15.46 ± 2.92	15.50 ± 2.97	463	5–22.5	114.22	< 0.001
Entrance	17.95 ± 0.89	16.20 ± 5.39	80	3.99–53.41	22.83 ± 9.59	21.21 ± 10.48	262	5.89–60.08	25.77 ± 10.62	24.74 ± 10.48	463	2.75–67.15	43.68	< 0.001
Crabs	3.81 ± 0.81	4.0 ± 0.0	21	2.0–6.0	12.48 ± 2.56	13.0 ± 2.97	21	8.0–16.0	22.14 ± 2.52	22.0 ± 2.97	21	18.0–27.0	53.82	< 0.001
Distance	1.45 ± 0.65	1.50 ± 0.74	80	0.3–3	7.17 ± 2.06	7.00 ± 2.22	262	2.5–13	7.99 ± 2.31	8.00 ± 2.97	463	2.5–14.5	227.6	< 0.001

The Kruskal–Wallace test was performed for standardized values.

Our hypothesis was tested using the Generalized Linear Mixed Model, which considered time of day (morning, noon, evening) and field study days as random factors.

**Figure 2:**
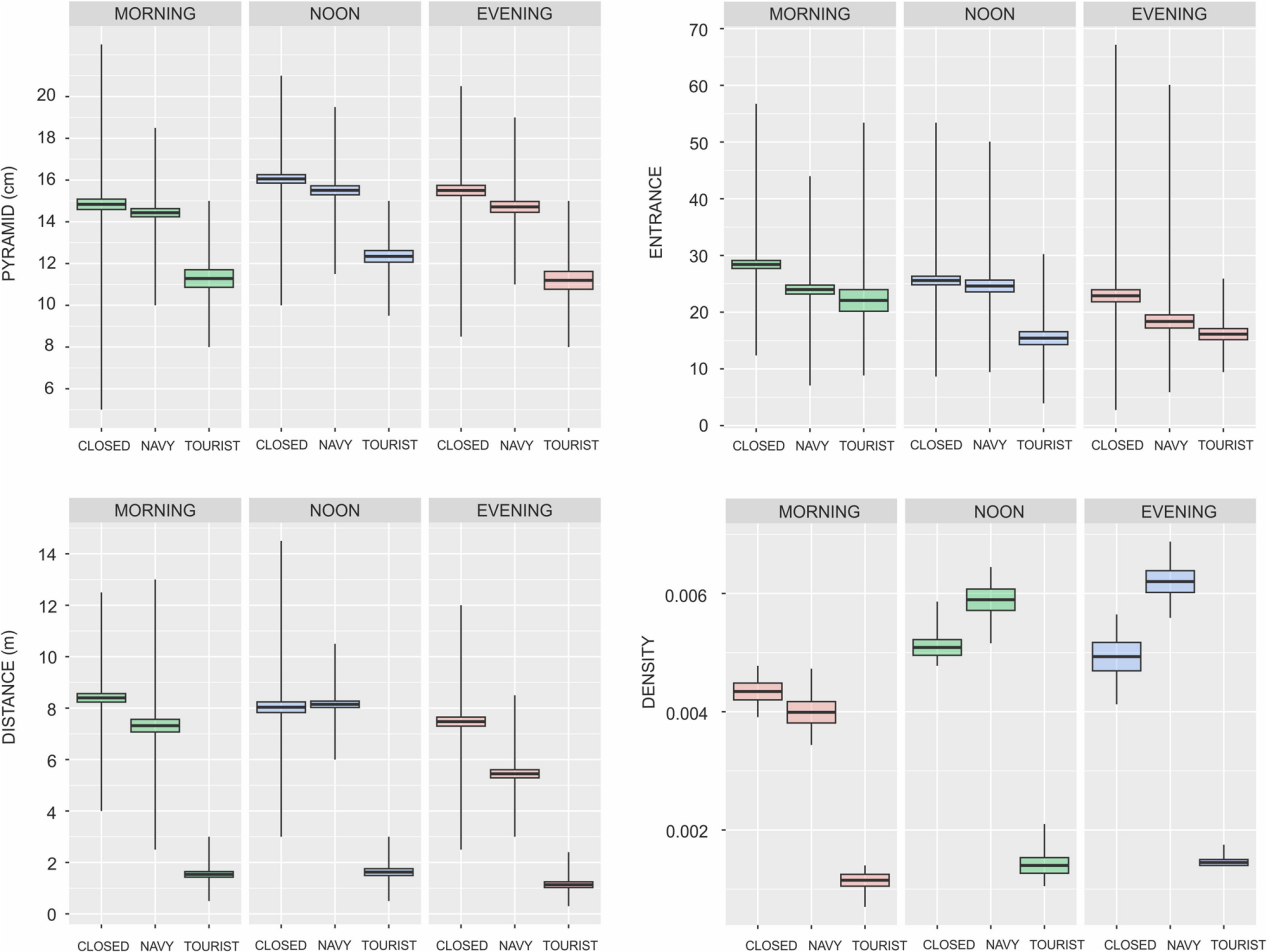
Differences in mean standardized value of PYRAMID, ENTRANCE SIZE, DISTANCE, and number of CRABS between beach types and time of day.

For the PYRAMID variable (pseudo-R^2^_conditional_ = 0.184, pseudo-R^2^_marginal_ = 0.090, Fig. 2), the GLMM indicated that the highest pyramids were observed on the closed section of the beach (Table 2). Contrasts for this model revealed no significant difference between the navy and tourist sections of the beach (Table 3, Fig S3A).

**Table 2:** The generalised linear mixed models model for difference between three section of beaches.

Variable	Estimation	SE	t-value	*p*
PYRAMID				
Navy-closed	− 0.476	0.121	− 3.924	< 0.0001
Tourist-closed	− 0.431	0.189	− 2.280	0.022
Random: study days	SD: 0.436			
Random: time of day	SD: 0.550			
ENTRANCE				
Navy-closed	− 0.112	0.032	− 3.516	0.0004
Tourist-closed	− 0.326	0.050	− 6.526	< 0.0001
Random: study days	SD: 0.335			
Random: time of day	SD: 0.152			
DISTANCE				
Navy-closed	− 0.865	0.157	− 5.480	0.0003
Tourist-closed	− 6.534	0.246	26.530	< 0.0001
Random: study days	SD: 0.340			
Random: time of day	SD: 0.671			
CRABS				
Navy-closed	0.099	0.044	2.238	0.02
Tourist-closed	− 1.282	0.044	− 28.928	< 0.0001
Random: study days	SD: 0.013			
Random: time of day	SD: 0.069

**Table 3:** Contrast analysis for generalised linear mixed models for differences between naval—tourist section of beaches.

Compared pair	Estimate	Se	t.ratio	*p*
PYRAMID				
Close—naval	0.4767	0.122	3.919	0.0003
Close—tourist	0.4313	0.189	2.279	0.0594
Naval—tourist	− 0.0453	0.199	− 0.228	0.9718
ENTRANCE				
Close—naval	2.970	0.750	3.967	0.002
Close—tourist	7.520	1.170	6.444	< 0.0001
Naval—tourist	4.550	1.230	3.705	0.0007
DISTANCE				
Close—naval	0.865	0.158	5.472	< 0.0001
Close—tourist	6.535	0.246	26.515	< 0.0001
Naval—tourist	5.670	0.259	21.860	< 0.0001
CRABS				
Close—naval	− 0.099	0.044	− 2.238	0.065
Close—tourist	1.282	0.044	28.928	< 0.0001
Naval—tourist	1.381	0.044	31.132	< 0.0001

Similarly for the ENTRANCE variable (pseudo-R^2^_conditional_ = 0.159, pseudo-R^2^_marginal_ = 0.051, Fig. 2) the GLMM showed a similar relationship, with the largest entrance to burrows observed on the closed section of the beach (Table 2). However, there was also difference between the tourist beach and the naval section (Table 3, Fig S3B).

The trend was also observed for the DISTANCE (pseudo-R^2^_conditional_ = 0.505, pseudo-R^2^_marginal_ = 0.436, Fig. 2) and DENSITY (pseudo-R^2^_conditional_ = 0.354, pseudo-R2_marginal_ = 0.303, Fig. 2) variables. The distance between burrows and the waterline was highest on the closed section (Table 2), and a difference was also found between the naval and tourist sections (Table 3, Fig S3C). Additionally, density was observed to be highest on the closed beach (Table 2), while it was statistically significantly lowest on the tourist section (Table 3, Fig S2D).

## Discussion

While we observed some variations between different times of day, our analysis confirmed our hypothesis that even minimal human intervention exerts pressure on RSGC number and activity. This underscores the importance of understanding the impact of human activities on endemic coastal species. Our study demonstrates that the previously described differences in crab behaviors in experimental setups, consisting two groups, the tourist beach and the naval beach^19^, are insufficient to fully comprehend the pressure exerted by humans on this endemic coastal species. This highlights the necessity for comprehensive studies that consider various factors, including beach usage patterns and levels of human disturbance, to accurately assess and address conservation challenges facing coastal species^33–35^.

Our findings revealed that the largest crabs, as indicated by the dimensions of their burrow entrances, and the tallest pyramids were observed on the closed section of beach. Furthermore, we also found that the burrows on the closed section were furthest from the waterline. The results suggest that larger individuals with superior competitive abilities dominate beaches with better habitat conditions, such as the closed beach section ^35^. This competition may force smaller individuals to occupy or migrate to parts of beaches subjected to tourism pressure. Moreover, smaller individuals may be compelled to keep their burrows closer to the waterline.

Alternatively, it is also plausible that smaller individuals observed on the tourist section are juveniles. Therefore, the segregation of individuals between beaches may not solely be due to competition but could also reflect different stages of individual development. Another possibility is that the distribution pattern is influenced by the variation in the required frequency of gill replenishment between adult and juvenile ghost crabs^36^.

Regardless of the underlying reasons for the differences in RSGC activity observed across beach sections, the closed beach serves as a crucial refuge from the stress caused by tourist activities, facilitating undisturbed reproduction. This sanctuary mitigates the impacts of trampling on burrows and pyramids, which are critical resources for shore crabs, especially *Ocypode* species^13–15,34^. Burrows have evolved into essential structures for these crabs, providing protection from predators, extreme temperatures, and desiccation/respiration, also serving as mating sites^13–15,33,37–39^. Similarly, pyramids play a significant role for the study species, serving as secondary sexual signals^13–15,17^. Therefore, destruction or alteration of these structures by tourists may lead to unnatural changes in sexual selection and higher energy consumption by individuals within this species^40^, with potential long-term consequences for population dynamics and ecosystem health.

The observed differences in the distance of burrows from the waterline between sections likely stem from individuals’ optimizing the time spent digging these structures. It is known that all RSGC burrows extend to the waterline at the bottom^11^. Thus, the further from the waterline, the more time an individual must invest in digging a sufficiently deep burrow. In the conditions of the tourist section of the beach, placing the burrow close to the waterline potentially shortens the time spent on this activity, allowing the accumulation of energy resources to repeat digging the burrow or rebuilding the conspicuous pyramid if destroyed by tourists^40^. Therefore, the choice of placing the burrow and pyramid is under strong natural selection pressure, reflecting the species’ adaptation to its environment.

Greater numbers of crabs were observed on the closed section of the beach; but we suspect that crabs may migrate from closed section to the tourist section to access food resources, which may include organic waste generated by increasing tourism activities. This highlights the complex interplay between resource availability and population dynamics in coastal ecosystems^40–42^, emphasizing the need for further research to better understand these relationships and inform conservation efforts.

The movement of individuals between beach sections subjected to various human pressures is a typical biological and ecological process with far-reaching implications. Animal movement plays a crucial role in regulating relationships within trophic networks and facilitating the transfer of organic matter between different habitats^39–43^.

The studied species, renowned for its remarkable mobility, particularly during nocturnal hours when it traverses distances of up to 300 m in search of food, demonstrates this phenomenon^43–46^. While our observations were conducted during the day, it is plausible to infer that individuals likely maintained fidelity to their respective territories during this timeframe, drawing from insights gleaned from prior behavioral research on the genus Ocypode.

During the day, foraging activities typically involve younger individuals, with adults displaying such behavior sporadically^44^. However, forced migration of adults during the day to undisturbed sections of the beach could occur due to sudden increases in tourist presence, potentially leading to nocturnal migration in the opposite direction, driven by the high availability of organic matter left by beachgoers (see Pittman and McAlpine^38^).

As a result, the population size of the studied system is highly dynamic at any given moment. A comprehensive understanding would necessitate marking each individual and conducting continuous 24-h observations and genetic population study^39^, which is not feasible in our case due to restrictions on access to closed sections of the beach.

Due to this restrictions, we were unable to determine the predators that may prey on RSGC. However, previous studies have shown that the genus Ocypode is vulnerable to predation by birds^46^, mammals^47^, reptiles^46^, fish^48^, and humans^49–52^. In our observations at the tourist beach, we observed instances of people killing crabs, as well as abandoned/wild dogs (*Canis lupus familiaris*), domestic cats (*Catus silvestris*), and the invasive house crow (*Corvus splendens*) preying on them. These animals have been documented to have negative impacts on entire coastal ecosystems^51–53^. Wild dogs, in particular, compete with native fauna for access to non-natural organic resources^54^. Thus, spatial isolation of RSGC by separating a small beach enclave could help prevent excessive predation by domesticated animals.

From a conservation biology perspective, it is important to note that Eilat is a heavily tourist-oriented city, with little consideration for environmental aspects in its infrastructure. The planned development of the entire Israeli section of Red Sea beaches to make them more tourist-friendly^55^ poses a significant threat to the RSGC population. Activities such as heavy machinery use on the shore, cement spillage on the beaches, and proximity to the waterline already raise major concerns. For instance, a contractor dumped a truckload of slate onto the study beach, as shown in Fig. 1. These uncontrolled and unregulated activities are likely to have far-reaching impacts on the conservation of the endemic RSGC and all other benthic aquatic and beach fauna^20^. Urgent measures are needed to mitigate these threats and protect the fragile ecosystems of the Red Sea beaches.

Due to the high ecological dynamics of the system we studied, it is crucial to acknowledge the methodological limitations we encountered. Chief among these is the relatively low replication of observations for such a dynamic system, which may lead to underestimations or distortions in our results. This concern is partly supported by the impact of random factors identified in our model. Furthermore, the frequent variations in human presence and activities on the beaches posed challenges to our attempts to accurately assess the width of each of the three designated sections. Notably, the usage pattern of the open beach by tourists exhibited significant irregularity, characterized by substantial daily fluctuations particularly attributable to camping activities. The naval beach area, constrained by fortifications erected along its perimeter, featured a narrow strip allocated for the storage of equipment essential for military exercises. Consequently, our approach to section demarcation had to be continually adjusted to accommodate the dynamic conditions encountered during each visit.

However, the results obtained align with those of our previous studies on the behavior of crabs on navy and tourist sections of the beach conducted several years earlier. This suggests that despite potential disruptions in the results due to the short observation period, the overall pattern described for the studied species remains consistent with reality. While these limitations warrant caution in interpreting our findings, they also underscore the need for further research to validate and expand upon our observations in order to better understand the dynamics of this coastal ecosystem.

Therefore, experimental setups testing the impact of human pressure on the genus Ocypode based on two groups (strong human pressure vs. moderate human pressure) are insufficient to fully demonstrate the actual impact of human activity on coastal ecosystems. Our research has revealed that even minimal human impact can induce changes in the behavior of these crustaceans, potentially affecting population dynamics.

The RSGC exhibits a dependency on organic resources left by beach visitors, thereby influencing its survival and habitat utilization significantly. Nonetheless, for its reproductive success, it necessitates access to undisturbed natural environments where natural selective pressures, such as competition and predation, prevail exclusively. Consequently, ensuring the preservation of isolated sections assumes paramount importance for the viability and potential expansion of this diminishing population throughout the Gulf of Eilat/Aqaba. In light of this, we advocate for a straightforward and economically viable strategy: the establishment of small, secluded beach sanctuaries along this gulf. We posit that this measure would mitigate the adverse impacts of tourism while concurrently fostering the well-being of various sandy beach species.

In summary, despite the logistical challenges, our study demonstrates that in the northernmost reaches of the eastern part of the Red Sea, a mosaic of three adjacent fenced sections of a beach remains for the endemic RSGC, with one intact part and two sections showing varying degrees of deterioration. Our analysis revealed distinct differences in the activity of the endemic ghost crab species between small isolated sections of the beach differs without human activity as compared to beach sections under tourist pressure. This finding is particularly significant considering the increasing tourism pressure on this ecosystem.

Our study suggests that separating a small part of the beach along the Red Sea could ensure the survival of not only ghost crabs but potentially other sandy beach species as well, without adversely affecting the tourist attractions of the resorts. This underscores the importance of conservation efforts aimed at preserving and protecting these critical habitats for the long-term sustainability of coastal ecosystems.

### Supplementary Information

Supplementary Information.

## Data Availability

The data is available directly from the author, Reuven Yosef, ryosef60@gmail.com.
