# Supplementary material for: No man’s land support the endemic Red Sea ghost crab (Ocypode saratan) in the Gulf of Eilat
Source: Sci Rep. 2024 May 31;14:12577. doi: 10.1038/s41598-024-63326-y (PMC11143321; doi:10.1038/s41598-024-63326-y)

**Material and methods**

Fig S1. Matrix correlation between explained variables

**
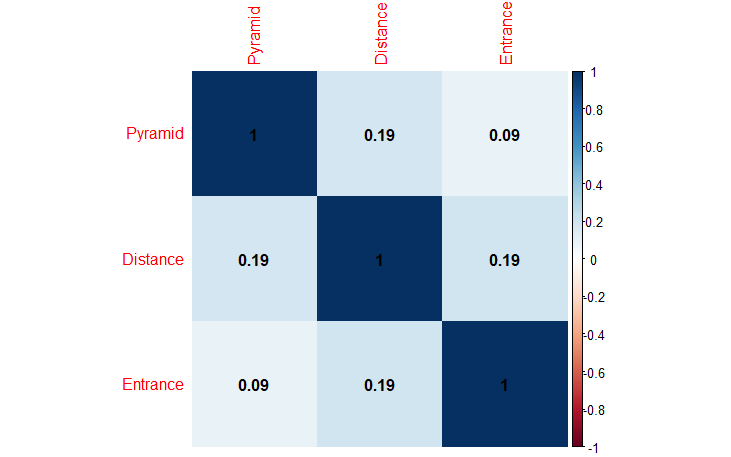
**

***Differences between inspection days for our explained variables***

Kruskal-Wallis rank sum test

data: data$**Pyramid** by as.factor(data$inspection_day)

Kruskal-Wallis chi-squared = 101.53, df = 7, p-value < 2.2e-16

Kruskal-Wallis rank sum test

data: data$**Entrance** by as.factor(data$inspection_day)

Kruskal-Wallis chi-squared = 49.808, df = 7, p-value = 1.575e-08

Kruskal-Wallis rank sum test

data: data$**Distance** by as.factor(data$inspection_day)

Kruskal-Wallis chi-squared = 24.626, df = 7, p-value = 0.0008839

Kruskal-Wallis rank sum test

data: data$**Crabs** by as.factor(data$inspection_day)

Kruskal-Wallis chi-squared = 0.60712, df = 7, p-value = 0.999

***Differences between time of day for our explained variables***

Kruskal-Wallis rank sum test

data: data$**Pyramid** by as.factor(data$Time.of.day)

Kruskal-Wallis chi-squared = 21.037, df = 2, p-value = 2.703e-05

Kruskal-Wallis rank sum test

data: data$**Entrance** by as.factor(data$Time.of.day)

Kruskal-Wallis chi-squared = 51.4, df = 2, p-value = 6.897e-12

Kruskal-Wallis rank sum test

data: data$**Distance** by as.factor(data$Time.of.day)

Kruskal-Wallis chi-squared = 33.615, df = 2, p-value = 5.019e-08

Kruskal-Wallis rank sum test

data: data$**Crabs** by as.factor(data$Time.of.day)

Kruskal-Wallis chi-squared = 8.9468, df = 2, p-value = 0.01141

Fig S2. Diagnostics of variables included in the model

1. Height of pyramid (HEIGHT) with **normal** distribution


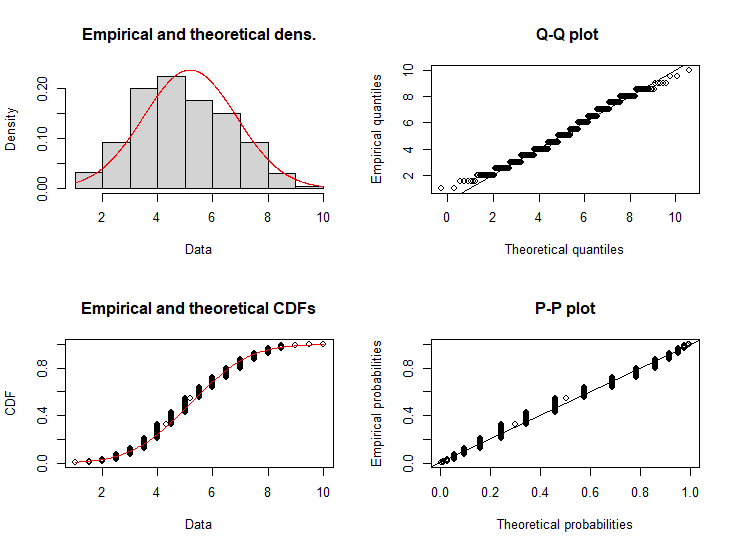


1. Distance between burrow and water line (DISTANCE) with **normal** distribution


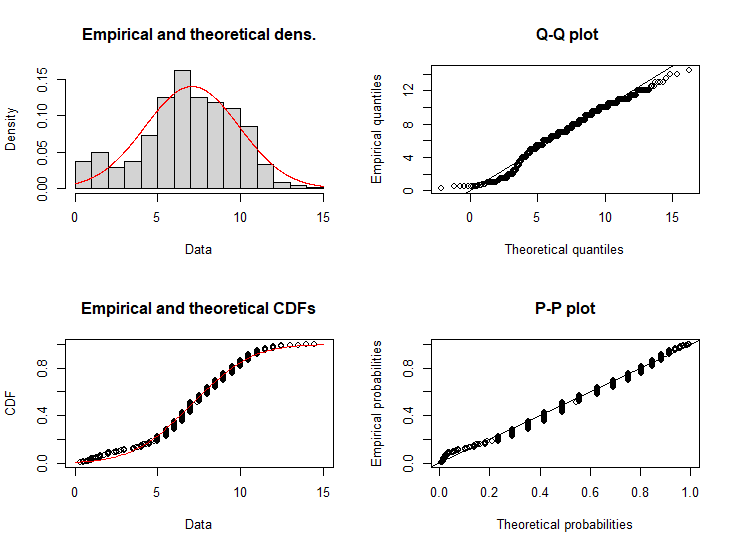


C) Area of entrance to burrow (ENTRANCE) with **gamma** distribution


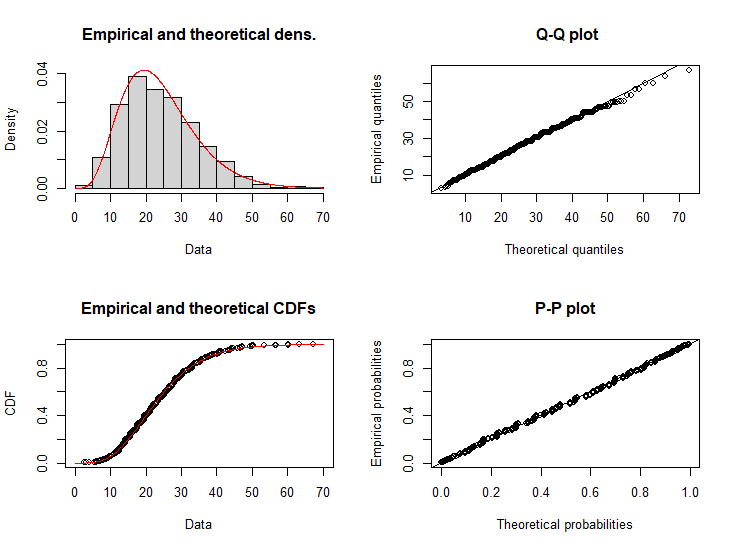


D) CRABS with **gamma** distribution


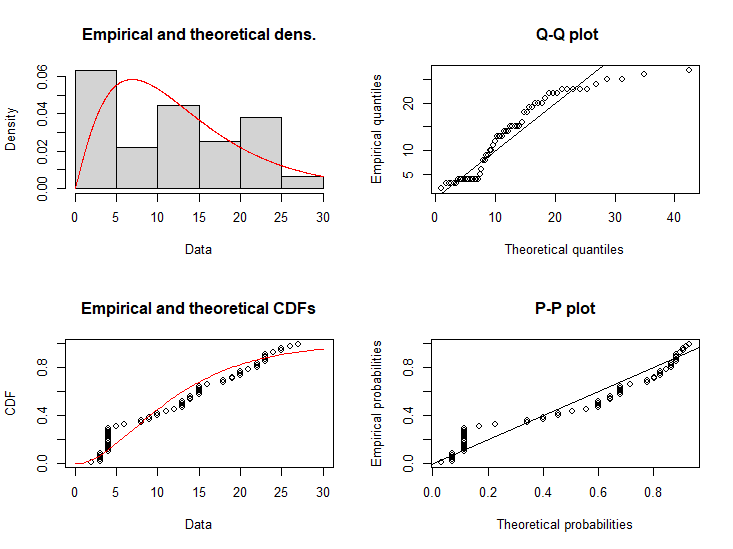


Fig S3. Contrast analysis plots

1. PYRAMID model


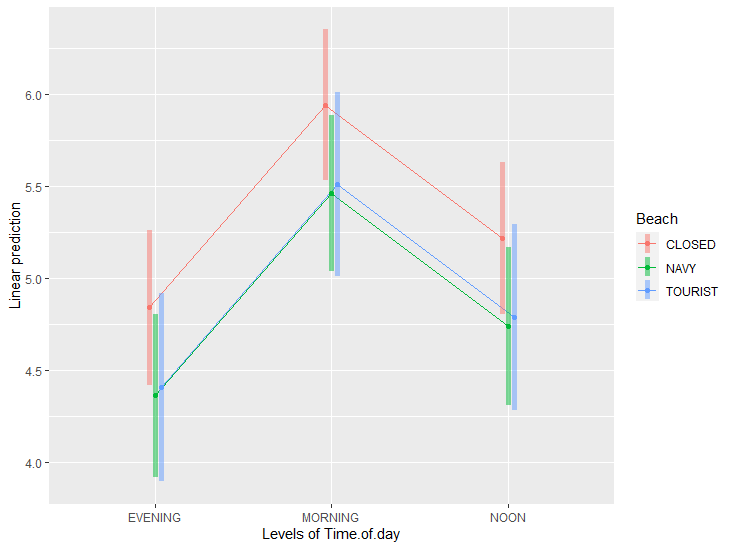


1. ENTRANCE model


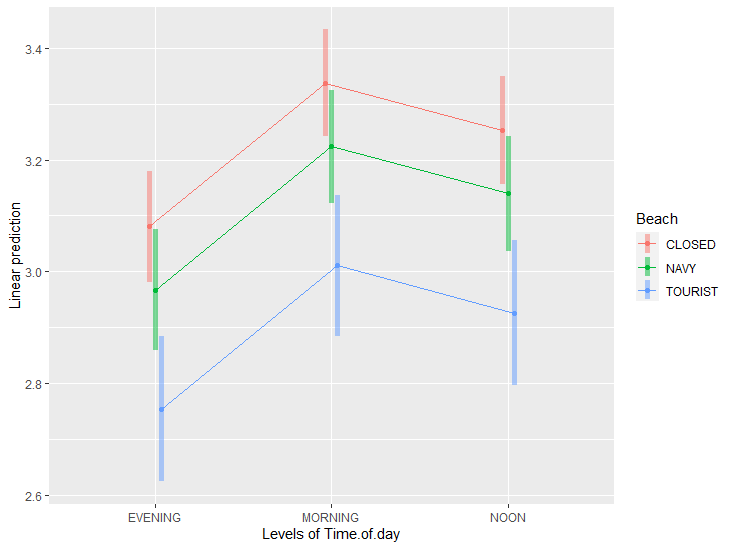


1. DISTANCE model


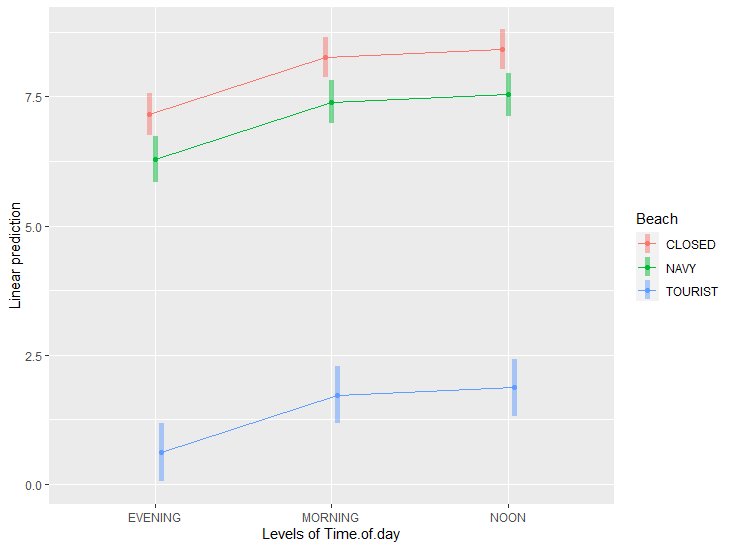


1. CRABS density model


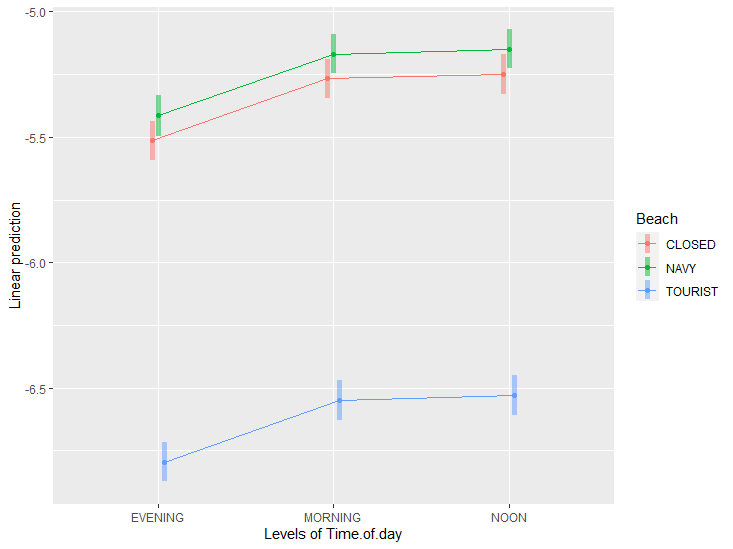

Supplement: Supplementary file 1 — Supplementary Information. [file 41598_2024_63326_MOESM1_ESM.docx]
